# Incidence of Gastrointestinal Stromal Tumors in the United States from 2001-2015: A United States Cancer Statistics Analysis of 50 States

**DOI:** 10.7759/cureus.4120

**Published:** 2019-02-22

**Authors:** Nicolas Patel, Bikramjit Benipal

**Affiliations:** 1 Internal Medicine, New York University School of Medicine, New York, USA; 2 Internal Medicine, Temple University, Philadelphia, USA

**Keywords:** gist, gastrointestinal stromal tumor, gastroenterology, epidemiology, cancer, incidence

## Abstract

Introduction

Gastrointestinal stromal tumor (GIST) was once a mislabeled cancer with inaccurate incidence data in the United States (US). Since the discovery of a gain of function mutation in kit, the proper identification of GIST has greatly improved. Given this inaccuracy in prior GIST incidence, our goal in this study was to evaluate the true incidence of GIST in at-risk populations in all 50 states.

Methods

The United States Cancer Statistics (USCS) was used to obtain data for GISTs from 2001 to 2015. Incidence analysis was done for sex, race, stage, primary location, and US regional location.

Results

The overall incidence of GISTs from 2001-2015 was 0.70 per 100,000 people per year. Overall incidence rates were greatest for each stratification of males, blacks, localized disease, primary location in the stomach, and the Northeast. The incidence in blacks increased with an annual percent change (APC) of 6.27 between 2001 and 2015. Between 2001 and 2015, the incidence of localized disease and GISTs with a primary location in the stomach increased with APCs of 8.90 and 6.25, respectively. In the Northeast, between 2001 and 2003, the incidence initially increased expeditiously (APC 13.35); however, after 2003, the incidence continued to rise but no longer at the same rapid rate (APC 3.05).

Conclusion

In our study, we investigated the incidence of GISTs using data from the USCS database for all 50 states in the US. We found an alarming rise in the incidence in blacks, localized disease, the stomach, and those in the Northeast. Ultimately, further studies are required to identify risk factors for the development of GISTs; however, our study will serve as a template to help guide those studies.

## Introduction

Gastrointestinal stromal tumors (GISTs) are uncommon mesenchymal cancers that occur most commonly in the gastrointestinal (GI) system [1]. In the past, GISTs were mislabeled as smooth muscle neoplasms and were diagnosed as leiomyomas, leiomyoblastomas, leiomyosarcomas or schwannomas [1, 2]. However, in 1998, the discovery of a gain of function mutations in kit, a mast/stem cell growth factor receptor, in GIST and associated kit-positive immunostaining gave objective data for the identification and proper diagnosis of GIST [3, 4]. Prior studies that have evaluated the incidence of GISTs have used the National Cancer Institute’s (NCI) Surveillance, Epidemiology and End Results (SEER) program. However, the SEER database only represents approximately 28% of the United States (US) population [5]. Consequently, the SEER database, although aimed to represent the entire US population, can misrepresent certain racial/ethnic groups and regions within the US [6]. The United States Cancer Statistics (USCS) database combines both the Centers of Disease Control and Prevention’s (CDC) National Program of Cancer Registries (NPCR) and the SEER database to include data on all 50 states and thus can have a far greater representation of the US population [7]. In this study, we evaluated the incidence of GISTs in all 50 states between 2001 and 2015 stratified by different risk factors.

## Materials and methods

Incidence data for GIST between 2001 and 2015 was obtained from the USCS database [8]. The USCS database provides official federal statistics on cancer incidence and population data for all 50 states and the District of Columbia [6]. Cases were selected by primary site: C15.0 cervical esophagus, C15.1 thoracic esophagus, C15.2 abdominal esophagus, C15.3 upper third of esophagus, C15.4 middle third of esophagus, C15.5 lower third of esophagus, C15.8 overlapping lesion of esophagus, C15.9 esophagus not otherwise specified (NOS), C16.0 cardia, C16.1 fundus of stomach, C16.2 body of stomach, C16.3 gastric antrum, C16.4 pylorus, C16.5 lesser curvature of stomach, C16.6 greater curvature of stomach, C16.8 overlapping lesion of stomach, C16.9 stomach NOS, C17.0 duodenum, C17.1 jejunum, C17.2 ileum, C17.3 Meckel's diverticulum, C17.8 overlapping lesion of small intestine, C17.9 small intestine NOS, C18.0 cecum, C18.1 appendix, C18.2 ascending colon, C18.3 hepatic flexure of colon, C18.4 transverse colon, C18.5 splenic flexure of colon, C18.6 descending colon, C18.7 sigmoid colon, C18.8 overlapping lesion of colon, C18.9 colon NOS, C19.9 rectosigmoid junction, C20.9 rectum, C21.0 anus, C21.1 anal canal, C21.2 cloacogenic zone, C22.0 liver, C22.1 intrahepatic bile duct, C23.9 gallbladder, C24.0 extrahepatic bile duct, C24.1 ampulla of Vater, C24.8 overlapping lesion of biliary tract, C24.9 biliary tract NOS, C25.0 head of pancreas, C25.1 body of pancreas, C25.2 tail of pancreas, C25.3 pancreatic duct, C25.4 islets of Langerhans, C25.7 other specified parts of pancreas, C25.8 overlapping lesion of pancreas, and C25.9 pancreas NOS. International Classification of Diseases (ICD) for Oncology, 3rd edition codes were used to extract data for gastrointestinal stromal sarcoma (8936/3). Incidence analysis was done for sex, race, stage, primary location within the gastrointestinal (GI) system, and US regional location (Northeast, Midwest, South, and West). Race included those that were white, black, and Asian or Pacific Islanders (API). Primary location within the GI system included the stomach, small intestine, and the colorectum. Incidence analysis used Tiwari et al., 2006 modifications for confidence interval (CI) [9]. The Joinpoint Regression Program (version 4.5.0.1, DigitCompass LLC, Maryland, USA) was utilized to create incidence graphs and calculate annual percent change (APC) using the least square method [10]. Incidences are per 100,000 and were adjusted to the year 2000 US standard population. For all analysis, p < 0.05 was considered statistically significant.

## Results

A total of 34,257 patients were included in the incidence analysis between 2001 and 2015 (Table 1). A total of 17,687 (51.6%) males and 16,570 (48.4%) females were included in the analysis. A total of 33,817 had an identifiable race at the time of diagnosis. Of those, 25,324 (74.9%) were white, 6,677 (19.7%) were black, and 1,816 (5.4%) were API. There were a total of 31,019 patients with a stage at diagnosis. Of those, 21,300 (68.7%) were localized, 4,485 (14.5%) were regional, and 5,234 (16.8%) were distant. There were a total of 33,823 patients with a primary site of disease. Of those, 22,023 (65.1%) were in the stomach, 10,158 (30.0%) were in the small intestine, and 1,642 (4.9%) were in the colorectum. There were a total of 34,257 patients with an identifiable region within the US at the time of diagnosis. Of those, 7,591 (22.2%) were in the Northeast, 7,289 (21.3%) were in the Midwest, 12,892 (37.6%) were in the South, and 6,485 (18.9%) were in the West.

**Table 1:** Patient Characteristics.

Patient characteristics		
Gender (n = 34,257)		
	Count	Percent
Male	17,687	51.6%
Female	16,570	48.4%
Race (n = 33,817)		
	Count	Percent
White	25,324	74.9%
Black	6,677	19.7%
Asian or Pacific Islander	1,816	5.4%
Stage (n = 31,019)		
	Count	Percent
Localized	21,300	68.7%
Regional	4,485	14.5%
Distant	5,234	16.8%
Primary location (n = 33,823)		
	Count	Percent
Stomach	22,023	65.1%
Small Intestines	10,158	30.0%
Colorectum	1,642	4.9%
Regions (n = 34,257)		
	Count	Percent
Northeast	7,591	22.2%
Midwest	7,289	21.3%
South	12,892	37.6%
West	6,485	18.9%

**Figure 1:**
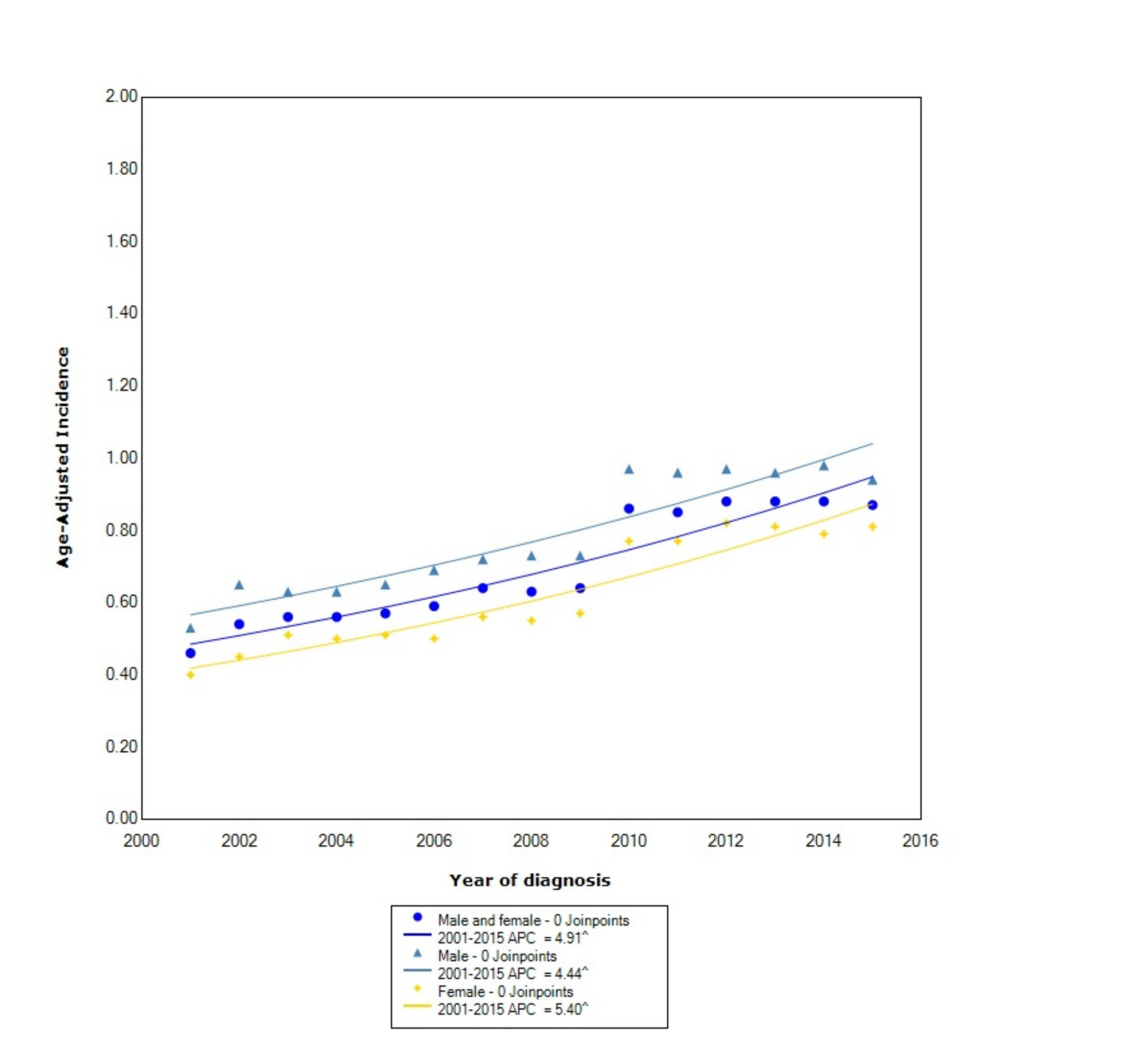
Incidence Rate, Sex. APC: Annual Percent Change ^ Indicates that the APC is significantly different from zero at the alpha = 0.05 Age-adjusted incidences are per 100,000 and age adjusted to the 2000 United States standard population.

The overall incidence of GIST from 2001 to 2015 was 0.70 per 100,000 people per year. Males had an overall incidence rate of 0.80 (95% CI: 0.78-0.81), which was greater than females who had an incidence of 0.63 (95% CI: 0.62-0.64). The incidence in males, between 2001 and 2015, increased at a statistically significant rate (APC 4.44). The incidence in females increased at a greater rate than in males with an APC of 5.40 (Figure 1).

**Figure 2:**
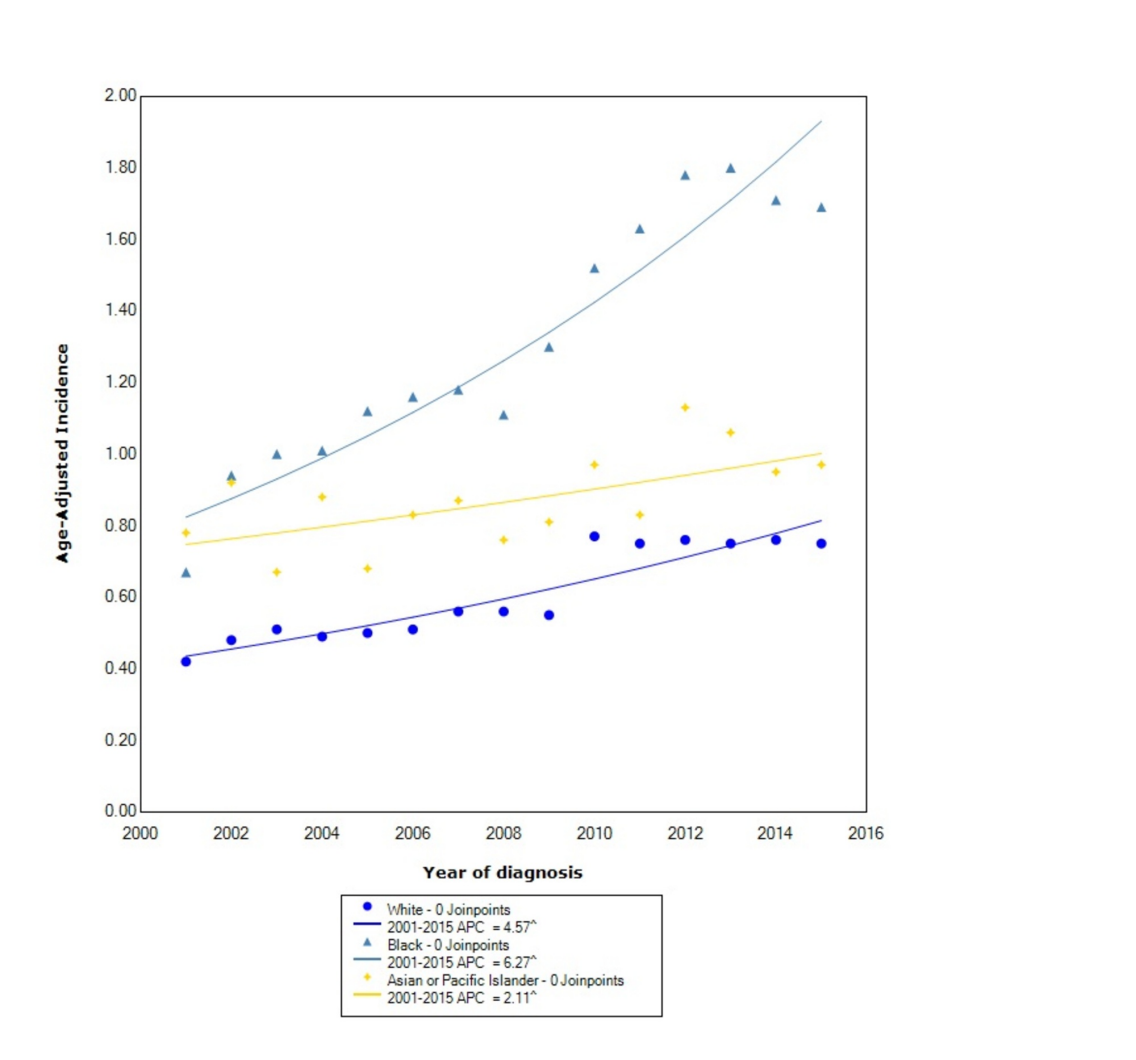
Incidence Rate, Race. APC: Annual Percent Change ^ Indicates that the APC is significantly different from zero at the alpha = 0.05 Age-adjusted incidences are per 100,000 and age adjusted to the 2000 United States standard population.

When stratified by race, GIST had the greatest incidence in blacks (1.35, 95% CI: 1.32-1.38) followed by API (0.89, 95% CI: 0.85-0.93) and lastly whites (0.62, 95% CI: 0.61-0.62). The incidence in blacks, between 2001 and 2015, increased at the greatest rate with a statistically significant APC of 6.27. The incidence in whites increased with a statistically significant APC of 4.57 between 2001 and 2015. The incidence in API also increased, between 2001 and 2015, with a statistically significant APC of 2.11 (Figure 2).

**Figure 3:**
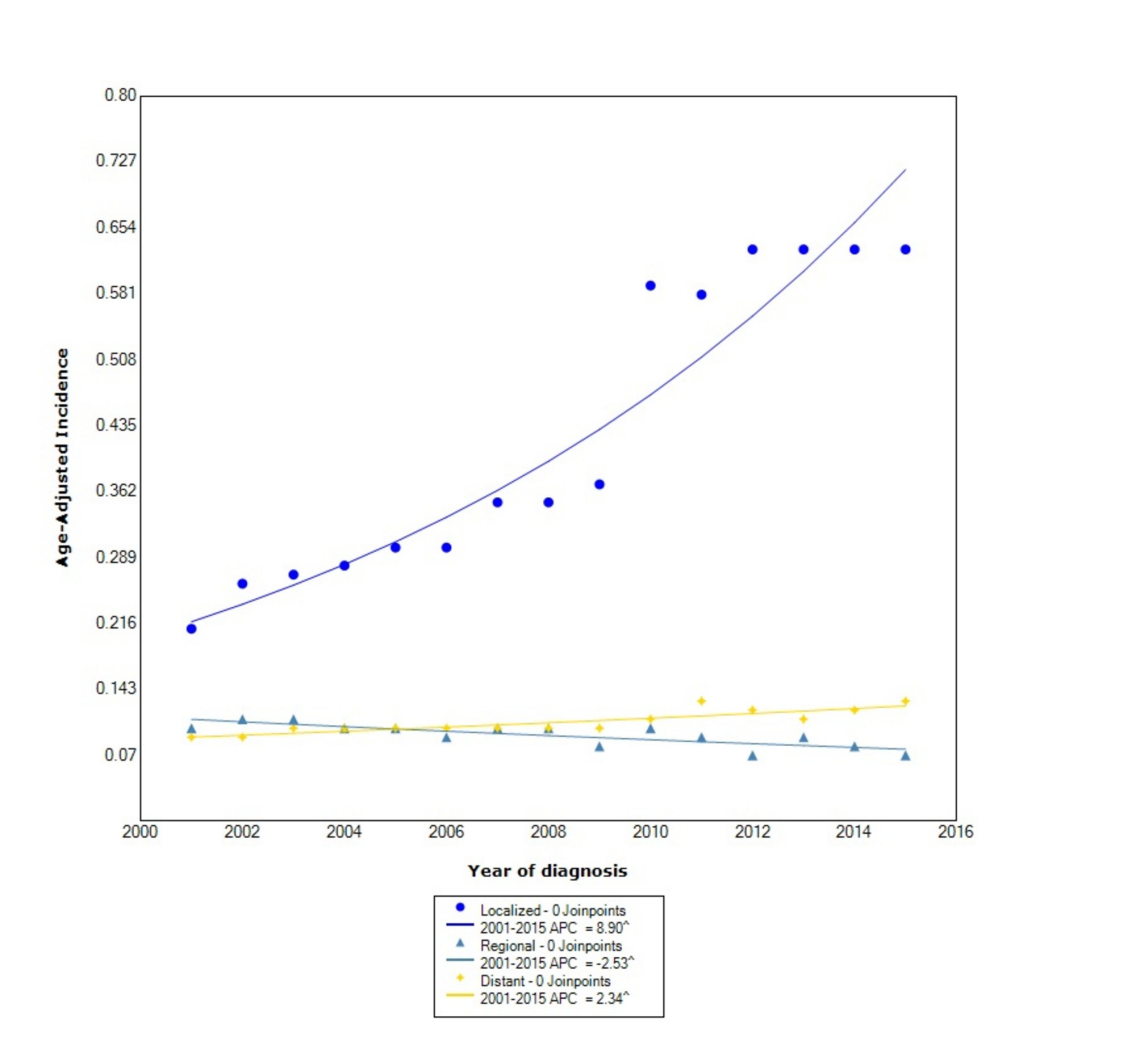
Incidence Rate, Stage. APC: Annual Percent Change ^ Indicates that the APC is significantly different from zero at the alpha = 0.05 Age-adjusted incidences are per 100,000 and age adjusted to the 2000 United States standard population.

When comparing stage at diagnosis, GIST had the greatest incidence in those with localized disease (0.44, 95% CI: 0.43-0.44), followed by distant disease (0.11, 95% CI: 0.10-0.11), and lastly regional disease (0.09, 95% CI: 0.089-0.095). The incidence of localized disease, between 2001 and 2015, increased at a rapid rate with a statistically significant APC of 8.90. The incidence of regional disease, however, decreased with statistical significance between 2001 and 2015 with an APC of -2.53. The incidence of distant disease during this time increased with statistical significance (APC 2.34) (Figure 3).

**Figure 4:**
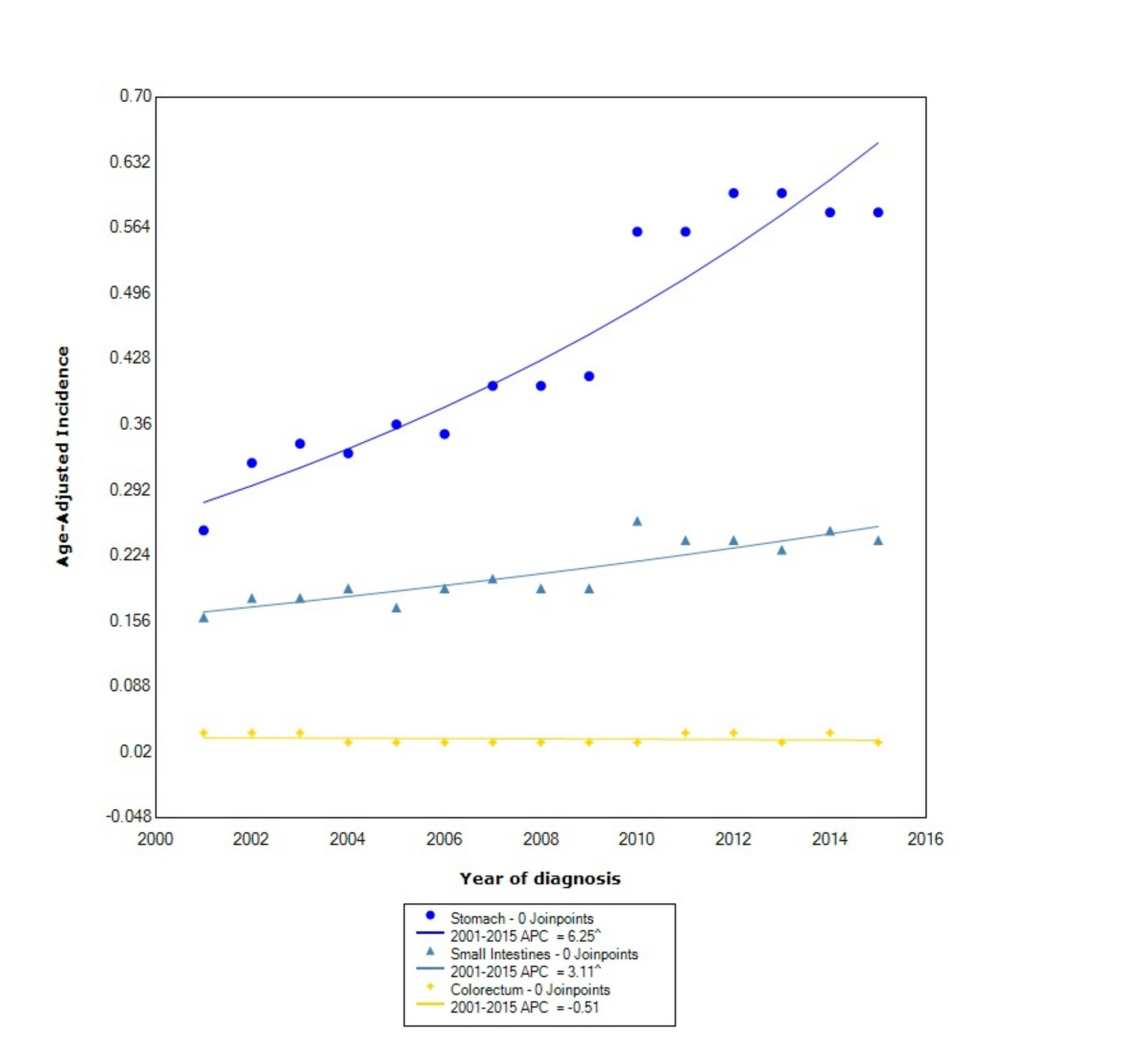
Incidence Rate, Primary Location. APC: Annual Percent Change ^ Indicates that the APC is significantly different from zero at the alpha = 0.05 Age-adjusted incidences are per 100,000 and age adjusted to the 2000 United States standard population.

When stratified by location within the gastrointestinal tract, the incidence was greatest in the stomach (0.45, 95% CI: 0.446-0.458), followed by the small intestine (0.209, 95% CI: 0.205-0.213), and lastly the colorectum (0.034, 95% CI: 0.032-0.035). During this time, the incidence of GIST in the stomach increased at the greatest rate with statistical significance (APC 6.25). The incidence in the small intestines also increased with statistical significance with an APC of 3.11. The incidence in the colorectum, however, decreased between 2001 and 2015 with an APC of -0.51 (Figure 4).

**Figure 5:**
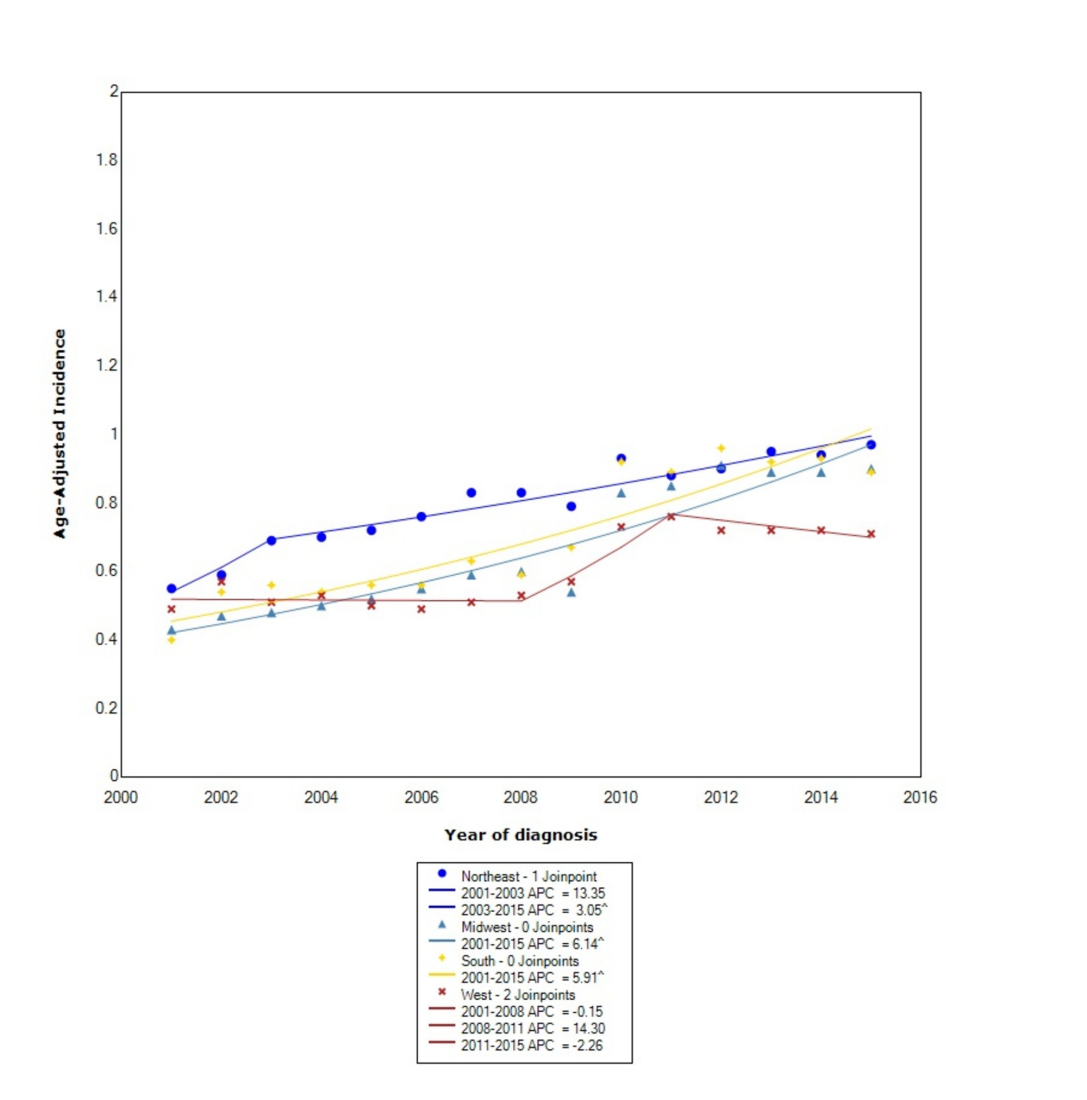
Incidence Rate, Region. APC: Annual Percent Change ^ Indicates that the APC is significantly different from zero at the alpha = 0.05 Age-adjusted incidences are per 100,000 and age adjusted to the 2000 United States standard population.

When stratified by regional location in the US, the incidence of GIST was greatest in the Northeast (0.81, 95% CI: 0.79-0.83), followed by the South (0.72, 95% CI: 0.71-0.73), the Midwest (0.67, 95% CI: 0.66-0.69), and lastly the West (0.61, 95% CI: 0.60-0.63). The incidence in the Northeast, between 2001 and 2003, increased at an expeditious rate with an APC of 13.35; however, after 2003, the rate continued to increase but no longer at the same rate (APC 3.05). In the South, the incidence continued to rise with statistical significance between 2001 and 2015 (APC 5.91). Between 2001 and 2015, the incidence in the Midwest increased with statistical significance (APC 6.14). In the West, between 2001 and 2008, the incidence was initially decreasing (APC -0.15), however between 2008 and 2011, the incidence started to rise at a rapid rate (APC 14.3). Subsequently, between 2011 and 2015, the incidence started to decrease again with an APC of -2.26 (Figure 5).

## Discussion

Our study, to the best of our knowledge, is the first to evaluate the incidence of GIST between 2001 and 2015 using the USCS database, which covers all 50 states. We found a difference in the incidence of GISTs for sex, race, stage, primary location, and US regional location. In our analysis, we found the overall incidence of GIST to be 0.70 per 100,000 people per year. There was an overall 1.27:1 male to female incidence ratio, which was similar to prior studies [11]. However, in contrast to prior studies, we found that the incidence, between 2001 and 2015, increased at a greater rate in females compared to males.

Similar to prior studies, we found that blacks had a greater incidence of GIST compared to other races [11]. However, we also found that the APC in blacks increased at a faster rate than other races. In the study by Ma et al., they found that APIs were nearly 1.5-times more likely to develop GISTs than whites [3]. We found that the overall incidence between APIs and whites is similar, however, contrary to prior findings, the incidence in whites increased at more than double the rate than in APIs. When comparing incidence by stage at diagnosis, we found that the overall incidence was greater in localized disease compared to regional and distant disease, which had similar overall incidences. However, we also found that the incidence of localized disease increased at over three times the rate as distant disease. Moreover, the incidence of regional disease has decreased. The incidence of GISTs, when stratified by primary location within the GI tract, is considerably greater in the stomach than other locations such as the small intestine or colorectum, which was also found by other studies [3]. We also found that the incidence in both the stomach and the small intestine is increasing between 2001 and 2015, whereas the incidence in the colorectum is decreasing. Moreover, the APC in the stomach is nearly double the APC of the small intestines.

Unlike prior studies, we took a novel approach to analyze the incidence of GISTs by US regional location. We found that the overall incidence, although similar, was slightly greater in the Northeast compared to other regions. Moreover, when we analyzed how the incidence changed over the years, we found that the incidence initially increased at an expeditious rate only in the Northeast. However, the incidence also increased at a substantial rate in the South and Midwest. Between 2001 and 2015, the West had a fluctuating incidence of GISTs, and after 2011 the incidence started to decrease again.

There were several strengths and limitations to our study. As the first study to use the USCS data to evaluate the incidence of GISTs in all 50 states, we were able to more accurately identify at-risk populations compared to prior studies. Other studies have used the SEER database which has data on a limited percentage of the US population and can underrepresent incidence patterns for cancers, unlike the USCS dataset [6]. Moreover, our study was done after the period of misdiagnosis of GISTs and thus represents a more accurate representation of incidence patterns compared to prior studies. A limitation of our study was the inability to stratify data by specific risk factors that may be contributing to the incidence patterns we found for the subpopulations we analyzed. Ultimately, further studies are needed to help determine correlations between specific risk factors and the results we found.

## Conclusions

In our study, we investigated the incidence of GIST in all 50 states. We found that the overall incidence was greatest in males, blacks, localized disease, the stomach, and those in the Northeast. The APC in these groups also increased at a rapid rate between 2001 and 2015. The reasons for these trends are still unclear and require further studies that look at specific risk factors and how they influence the incidence of GIST in specific stratifications. Our study, to the best of our knowledge, is the first study to investigate the incidence of GIST in all 50 states using the USCS database. Our findings will undoubtedly aid in the development of surveillance guidelines that can help reduce the morbidity and mortality of this cancer.
